# Epidemiology of hypertension and its relationship with type 2 diabetes and obesity in eastern Morocco

**DOI:** 10.1186/2193-1801-3-644

**Published:** 2014-10-30

**Authors:** Abderrahim Ziyyat, Noureddine Ramdani, Nour El Houda Bouanani, Jean Vanderpas, Benyounès Hassani, Abdeslam Boutayeb, Mohammed Aziz, Hassane Mekhfi, Mohammed Bnouham, Abdelkhaleq Legssyer

**Affiliations:** Laboratoire de Physiologie et Ethnopharmacologie, Département de Biologie – Faculté des Sciences, Université Mohamed Premier, B.P. 717, Boulevard Mohamed VI, Oujda, 60000 Maroc; Medical Microbiology Laboratory, Communicable and Infectious Diseases, Institut Scientifique de Santé Publique, Rue Engeland 642, 1180 Bruxelles, Belgique; Médecin endocrinologue diabétologue, Bd. Mohamed Abdou Imm. Sâada 1er étage, Oujda, Maroc; Laboratoire de modélisation stochastique et déterministe, Univ Mohammed I, Fac. Sciences, Oujda, Maroc

**Keywords:** Hypertension, Type 2 diabetes, Obesity, Abdominal obesity, Eastern Morocco

## Abstract

The major objectives of this work are to estimate the hypertension (HT) frequency in the east of Morocco and to study the relationship between HT, type 2 diabetes and obesity. Our sample is composed of 1628 adults aged 40 years and older, recruited voluntarily by using the convenience sampling method through 26 screening campaigns in urban and rural areas of the east of Morocco. We enumerated 516 hypertensive people (31.7%), without significant difference between women (32.5%) and men (30.2%). The known hypertensive people represent 10.1% of the whole sample. The frequency of HT, increases with age and it is more marked in rural (39.9%) than in urban areas (29%) (p < 0.001). It is significantly very high in diabetic subjects (69.9%) than among the non-diabetic ones (27.4%) (p < 0.001). The odd ratio (OR) of the diabetics to HT is 6.16 (IC95% [4.33-8.74]). Among the obese persons, HT is present at (40.8%) vs. (30.2%) among the subjects of normal weight (p < 0.05). The OR of the obese to HT is 1.6 (IC95% [1.26 - 2.04]). In conclusion, our results show a high frequency of HT in the east of Morocco; it affects nearly one third of the adult population aged 40 years and older. The relations between type 2 diabetes and obesity have also been identified and estimated.

## Introduction

Hypertension (HT) is a silent disease that affects the cardiovascular system and settles down without pain and symptoms at the beginning; therefore, it is bad or late diagnosed (WHO 2012; Nejjari et al. 2013). HT affects various organs and engenders grave affections; such as vascular cerebral accident and heart disorder (Nejjari et al. 2013). When the hypertensive patients are bad treated or untreated, the disease reduces the flexibility of arteries, what favors the apparition of the cardiac insufficiency, angina pectoris, atherosclerosis, stroke cerebral, microvascular diseases of retina and glomerulus (UKPDS 1998; Cohuet and Struijker-Boudier 2006). The management of the HT becomes more difficult when it is accompanied by diabetes and obesity (Garcia et al. 2008). The relations between these three affections are strong and well demonstrated (UKPDS 1998; Grossman and Messerli 1996; Hayashi et al. 2004). Multiple factors such as age, sex, stress, residence, physical inactivity and dietary habits are associated with this vascular disease (Joffres et al. 1997; Tazi et al. 2003; Karppanen and Mervaala 2006; Rosenthal and Alter 2012).

Regarding the epidemiology of HT, its prevalence is very worrying; the recent report of the WHO (WHO 2012) shows that one of three adults in the world is affected by HT, more than 2 billion people, most of whom are not diagnosed (Nejjari et al. 2013). In Africa, its prevalence exceeds 40% among adults in many countries often undiagnosed (Nejjari et al. 2013). At The Grand Maghreb, an international epidemiological, multicentric and transversal study conducted among patients who consult doctors in Algeria, Tunisia and Morocco, has reported an overall prevalence around 45.4% (Nejjari et al. 2013). In 2000, the ministry of health in Morocco published a prevalence of HT around 33.6% (30.2% for men and 37.0% for women) among a population aged more than twenty years (Tazi et al. 2003). To our knowledge, there are no field studies conducted in eastern Morocco of the prevalence of this disease except the work published by our team on the phytotherapy of HT and diabetes in 1997 (Ziyyat et al. 1997).

This study is considered as the follow-up and the continuity of a previous and original work carried out in the east of Morocco, concerning the screening of obesity, HT and mainly type 2 diabetes. The first results of the diabetes and obesity have been published in 2012 (Ramdani et al. 2012).

The main objectives of this study are to estimate the HT frequency among a population over the age of 40 years, living in the rural and urban regions of the east of Morocco, and to study the existing relations between this vascular pathology, type 2 diabetes and obesity taking into account the other intervening factors.

## Methods

### Sample

It is necessary to note that the subjects constituting this sample are the same individuals of the aforementioned work, which aimed mainly to estimate the frequency of type 2 diabetes. For that reason, two criteria have been set for the individuals to take part in this study: a minimum age of 40 years old and being fasting. The subjects had been previously informed of this study and they had voluntarily agreed to participate. They were recruited during the screening and prevention campaigns organized in collaboration with the health organizations and associations of civil society. They came mainly to test their blood sugar levels, their blood pressure and to identify their weight profile (Ramdani et al. 2012).

For practical reasons and especially sociocultural ones, it was difficult to take anthropometric and glycaemic measures for women in their homes while men were not there. To overcome this problem which prevented us from ensuring a random sampling, we opted for the convenience sampling method. This method has been detailed and discussed in the first article (Ramdani et al. 2012).

### Survey and questionnaire

The survey was conducted by a group of researchers trained in our laboratory. The realization of this work passes into four steps:

1. Demographic data;
2. Anthropometric data;
3. Risk factors data;
4. Measurement of arterial pression and glycemia.

### Diagnosis criteria of HT, diabetes and obesity

HT is considered if the systolic blood pressure (SBP) is greater than 140 mmHg or if the diastolic blood pressure (DBP) is higher than 90 mmHg (Mancia et al. 2007).

Diabetes (DM) is reported when the fasting blood glucose exceeds 1.26 g/l (Alberti et al. 1998).

Obesity is defined by a Body Mass Index BMI ≥ 30 kg/m^2^ (WHO 2003).

For abdominal obesity, it is considered in two ways (WHO 2003):

*Waist measurement (WC) ≥102 cm among men and ≥88 cm among women.

*Waist-to-hip ratio (W/H) above 0.8 for women and 1.0 for men.

### Statistics

Data analysis was carried out in the unit of Epidemiology at Brugmann University hospital (Brussels, Belgium) using SPSS software version 17.0. The continuous variables are presented as the means ± SD. The comparison of averages was performed with the test Z. The chi-square test was used to look for the dependency between the categorical variables. The univariate analysis has served in the study of binary relations between two variables. The odd ratio (OR) was used to measure the force of association between them. An (OR > 1) was considered as a risk factor when p < 0,05. To elucidate the relationship between several variables at the same time we made a multivariate analysis by logistic regression; the variables selected were those that had statically significant effects (p < 0.05).

The sample size was calculated by the formula: *n* = *p*(*1*-*p*) *X Z*^*2*^/*i*^*2*^; where 'n' is the expected size of the sample; 'p' is the estimated proportion of diabetes among the Moroccan population (6.6%) according to ministry of Health (Tazi et al. 2003); 'Z' is the critical value (percentile) for the standard normal distribution (1.96 for a 95% confidence interval CI95); and 'i' is the acceptable margin of error. For more security, we preferred to work with an interval instead of a fixed value. In our case "i" is between 1 and 2%. The calculations have led to numbers contained between 600 and 2300 subjects. We integrated initially 2027 detected people, after applying the inclusion and exclusion criteria mentioned above, the final sample took a final size of 1628 cases,

### Ethics

See the first article (Ramdani et al. 2012).

### Limits

See the first article (Ramdani et al. 2012).

## Results

**Figure 1:**
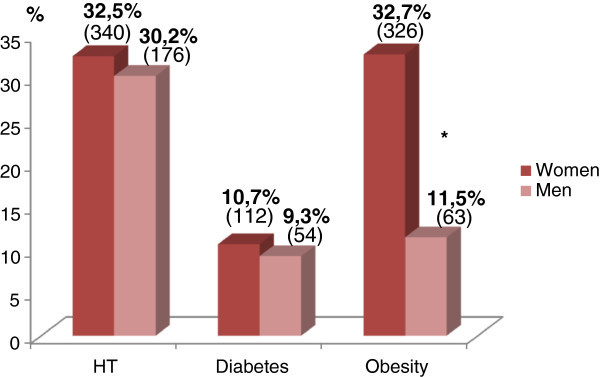
**Proportions and corresponding numbers (in parentheses) of HT, diabetes and obesity among women and men.** (Reminder: 80 data of BMI are missing). *p (F/H) < 0,001.

Our sample consists of 1628 subjects, the majority of them are women 63.4% compared to men 36.6% (p < 0.001). The subjects in urban regions constitute a number of (1224) individuals; the rest (404) live in rural regions (p < 0.001). The frequencies of HT, diabetes and obesity are presented in Figure 1. We remind that our database contains the measures of blood pressure and blood glucose of 1628 screened subjects; also, we signal that 80 data concerning the anthropometric measures are missing.

### Arterial hypertension in the total sample

The means and the values [min and max] of the SBP and the DBP are respectively 130.2 ± 20.3 mm Hg [80-230] and 80.1 ± 10.2 mm Hg [50-160]). ''120'' and ''80'' mm Hg are the values modes of SBP and DBP respectively. 65.4% of 1628 screened subjects were with a normal blood pressure, 2.9% were hypotensive, and 31.7% were hypertensive without significant difference between women 32.5% and men 30.2%. It should be noted that only 164 people (31.8%) from 516 hypertensive are aware of their disease (HT). Concerning the age factor the hypertensive subjects have the highest mean of age (58.2 years old) compared with normotensive (52.3 years old) and hypotensive people (50.4 years old). Regarding the residence factor, the frequency of HT in the rural regions was higher (39.9%) than in urban ones (29%) (p < 0.001).

26% of all screened subjects didn't know if HT existed among their families. 35% of the hypertensive patients confirmed the presence of HT in at least one member of their families (ascendants or collaterals) versus 25.9% among normotensive subjects; the difference is significant (p < 0.05).

### HT and diabetes

We should remind that in this investigation, the frequency of type 2 diabetes in the east of Morocco has been 10,2% without significant difference between women (10.7%) and men (9.3%) (Ramdani et al. 2012).

The average SBP is high in diabetics (140.3 ± 20.6 mm Hg) compared with non diabetics (130.1 ± 20.2 mm Hg) (p < 0.05). For the DBP we have not noted any significant difference (80.1 ± 10.2 vs. 80.3 ± 10.3 mm Hg).

As for the relationship between HT and diabetes, we have found that 22.5% of all 516 hypertensive patients were diabetics against 4.5% among the non hypertensive subjects (p < 0.001). On the other side, HT is significantly frequent among diabetics 69.9% compared with non diabetic subjects 27.4% (p < 0.001). The OR of the diabetics for HT is 6.16 (CI 95% [4.33 to 8.74]).

When considering the sex factor, we have found that among the 112 diabetic women, 73.2% were hypertensive vs 27.7% among non diabetic counterparts. Among the male sex, the situation is similar: 63% of all 54 diabetic men are hypertensive compared to 26.8% of those who are non diabetics (Table 1). The Khi-2 test has confirmed this association between HT and diabetes of the two sexes (p <0.001); thus the OR for HT is of 7.1 (CI95% [4.59 to 11.12]) and 4.63 (CI95% [2.58 to 8.31]) among diabetic women and men respectively.

**Table 1:** **Proportions and corresponding numbers** (**in parentheses**) **of women and men with HT in diabetics and non diabetics**

	Total of hypertensive (516)		Women hypertensive (340)		Men hypertensive (176)		
	Number	%	Number	%	Number	%	P (W/M)
Diabetics	(116)	69,9%	(82)	73,2%	(34)	63%	<0,05
Non diabetics	(400)	27,4%	(258)	27,7%	(142)	26,8%	NS
*p* (*diab*/*Non diab*)	<0,001		<0,001		<0,001		——

Finally, integrating the residence parameter, we have found that 25.1% and 16.8% of urban and rural hypertensive, respectively, are diabetics; the difference is very significant compared to the non hypertensive subjects. The OR for HT in diabetic patients living in the rural is 9.59 (CI95% [3.6 - 25.48], it is higher than that of patients living in the city: 6.13 (CI95% [4.17 - 8.99]).

### Hypertension and obesity

In this survey, the frequency of obesity in the east of Morocco was 25.1% with significant differences between women (32.7%) and men (11.5%) and between the urban regions (27.7%) and the rural ones (17.7%) (Ramdani et al. 2012).

In general, the average SBP is high among the obese (138.0 ± 20.4 mm Hg) compared with non obese (130.0 ± 20.3 mm Hg) (p < 0.05). For the DBP, we have not noted any significant difference (80.4 ± 10.3 vs. 80.2 ± 10.2 mm Hg).

40.6% of 389 obese are hypertensive vs 30.2% among 1159 non obese (p < 0.05). The OR for HT in obese subjects is 1.6 (CI95% [1.26-2.04]).

When we integrate the sex factor we find that 38.4% among the 326 obese women and 51.6% among the 63 obese men are hypertensive vs 31.4% and 28.6% among non obese women and men respectively; the differences are significant (Table 2). The chi-square test confirmed the association between these two disorders in both sexes. Thus, the OR of obese women and men for HT is of 1.4 (CI95% [1.0-1.8]) and 2.7 (CI95% [1.6-4.6]) respectively.

**Table 2:** **Proportions and corresponding numbers for women and men with HT in obese and non obese**

	Total of hypertensive (507)		Women hypertensive (336)		Men hypertensive (171)		
	Number	%	Number	%	Number	%	P (F/H)
Obeses	(157)	40,6%	(125)	38,4%	(32)	51,6%	<0,001
Non obeses	(350)	30,2%	(211)	31,4%	(139)	28,6%	NS
*p* (*Ob*/*Non ob*)	<0,05		<0,05		<0,001		——

(Reminder: 80 data of BMI are missing).

When considering the residence factor, we don’t find any significant differences between the frequency of HT among obese subjects in urban regions (38, 6%) and those in rural ones (45.6%), and between the non obese in urban regions (34.2%) and those in rural ones (38.5%).

## Discussion

### Frequency of HT

In our study, the frequency of hypertension (31.7%) was similar to that found in a national survey by the Ministry of Health (33.6%) (Tazi et al. 2003) and that found in the region of Tlemcen (32.7%) (Boukli Hacène and Meguenni 2007). However, we worked on a population aged 40 and over, while in the national survey, age was set at 20 years and older. We believe that our result is more realistic given that hypertension is rare in the age group 20-40 years. In addition, our study was conducted in a particular region that is a border area with Algeria to the east and with the enclave of Melilla in the north. Therefore, the black market is widespread and the area receives various products including food products from Europe, Asia, Africa, etc… This can have a direct influence on eating habits and consequently on the health of the citizens of this region.

Moreover, the results we found are not quite the same as the national survey; for example, in our study, the frequency of hypertension shows no significant difference between men (30.2%) and women (32.5%) while in the national survey, it is significantly higher among women (37% vs 30.2) (see other intervening factors). Moreover, in our study the frequency of obesity is much higher among women (32.7%) compared to that found in the national survey (22%).

### Other intervening factors

***Sex***: The frequency of HT among women (32.5%) is slightly higher than it is among men (30.2%), but without significant difference. In the national survey of the health ministry, women are more affected by HT (37%) than men (30.2%) (Tazi et al. 2003). The impact of sex on HT is highly controversial. Indeed, the work of Barrios and colleagues showed that women, for unknown reasons, are at greater risk of hypertension (Barrios et al. 2008). In a survey conducted in France in 2007, it is quite the opposite: the frequency of HT was higher in men (34.1%) than in women (27.8%) (Godet-Thobie et al. 2008).

***Residence***: Regarding this parameter, our results do not differ from the general trend of HT in urban and rural regions in Morocco. Thus, its frequency is significantly higher in rural regions (39.9%) compared with urban regions (29.04%). In Morocco, the official prevalence of HT in the rural is 43.3% vs 32.6% in the city (Tazi et al. 2003). Tazi explained this difference by the effect of stress in the rural life, especially in times of drought which sometimes lasts for successive years. The relationship between stress and HT is well documented (Rosenthal and Alter 2012).

In our situation, we can also talk about the impact of diet with emphasis on the too salty nature of many traditional dishes very well known in the countryside of eastern Morocco. We cite for examples some dishes as they are named in this region: Guéddid (pieces of meat cut into long, salted and dried in the sun), Smen (traditional salted butter) and Lakhliae (meat cooked in fat and salt). Indeed, these dishes are very rich in salt used for food preservation, since refrigerators are not yet widely available in rural areas and lack of electricity in some rural areas. The relationship between hypertension and sodium-rich diet is confirmed (Karppanen and Mervaala 2006; Vargas Alarcón 2006).

Finally, we can also mention the factor of consanguinity (Vargas Alarcón 2006), since rural areas and villages in eastern Morocco especially the southern part, are known by marriage within the tribe and cousins of the same family. The analysis of this factor in our database shows that 48.8% of hypertensive patients in rural areas have reported the presence of hypertension in the family against only 33% in urban areas; the difference is significant. In the literature, consanguinity and genetic factors are important parameters both of which contribute to the development of hypertension (Vargas Alarcón 2006).

***Age***: The frequency of HT in our sample increases with age in both sexes. Thus, we move from 23.4% and 9.7% in the first group [40-49 years old] to 52.6% and 51.5% for subjects aged over 70 years among women and men respectively. The increase of the frequency of HT along with age was known (Renzaho et al. 2014), and was also mentioned in the official report of the Ministry of Health on HT in Morocco (Tazi et al. 2003). This association is logical, because the human body accumulates, over time, the risk factors of HT.

### Relationship between HT and diabetes

22.5% of all 516 hypertensive patients are diabetics, vs only 4.5% among normotensive subjects. On the other side, 69% of the diabetics are hypertensive, vs 27.4% among non diabetics. The differences are highly significant in both cases. The risk of diabetics for HT is very high; OR = 6.16 (CI95% [4,33-8,74]). These results confirm the findings in the studies on the high incidences of HT in diabetic patients and vice versa, either in Morocco (Berraho et al. 2009), or in the world (UKPDS 1998; WHO 1985; Sowers 2003). The frequency of diabetes coexisting with hypertension is increasing in most developing and industrialized countries; for example, in the United States, the hypertension has been involved in 4.4% of deaths of diabetics, and diabetes was implicated in 10% of deaths of hypertensive persons. (The National High Blood Pressure Education Program Working Group 1994).

Regarding sex factor, we have found that the OR for HT is 7.1 (95% CI [4.59-11.12]) among diabetic women and 4.63 (CI95% [2.58-8.31]) among diabetic men. The relationship between type 2 diabetes, HT and sex factor has been studied and debated for a long time (WHO 1985; Stamler et al. 1993). Some studies indicate that diabetic women are more exposed to HT as it is the case in this work (Howards et al. 2006; Fidiarivony et al. 2010), it may be due to obesity factor, since we have found that they are more affected by excess weight. The search early of hypertension should be a habit of follow up of diabetics.

### Relation HT and obesity

The OR of obese subjects for HT is 1.6 (CI95% [1.26-2.04]). These results are consistent with several studies showing a high incidence of HT in obese persons (Jordan et al. 2007; Hall et al. 2001; Maniecka-Bryla et al. 2011). Physiologically, activation of the sympathetic nervous system, renal retention and activation of the renin-angiotensin system sodium seem to have a common point in the different diagrams proposed to explain this association (Vargas Alarcón 2006; Hall et al. 2001; Da Silva et al. 2009; Jansen et al. 2010).

Regarding the sex factor we find that obese men are more exposed to HT than obese women. To explain this, we have introduced the parameter of abdominal obesity, because several studies suggest its importance as a major risk factor for HT and cardiovascular diseases (Maniecka-Bryla et al. 2011; Gus et al. 2004; Janssen et al. 2004; Chuang et al. 2006). Indeed, the results of the comparison have showed that obese men have an average of waist circumference (113.8 ± 9.2 cm) and the ratio 'W/H' (1.22 ± 0.07), they are significantly higher than those found in obese women (104.3 ± 12.4 cm and 0.91 ± 0.08).

The disturbance in plasma concentrations of adipokines (especially the reduction in adiponectine), an enzyme secreted by the adipose tissue will play the role of hormonal signalling of inflammatory processes and contribute to the occurrence of insulin resistance and the dysfunction of the endothelium by a deficit of production of Nitric oxide ‘NO’ (Aprahamian and Sam 2011). This hypo adiponectinemy is considered as a risk factor for coronary artery disease and HT (Garcia et al. 2008; Aprahamian and Sam 2011).

### Relation between HT, diabetes and obesity

To elucidate this relation in our sample, we conducted a multivariate analysis by logistic regression. HT is chosen as a dependent variable. At first, we included all variables known by the interaction with HT: sex, age, residence, diabetes, BMI, WC and ratio W/H. After the calculations and elimination of variables without significant effect, we have identified three selected factors: diabetes with an OR = 6,6 (CI95% [3,52-9,05]); obesity (calculated by BMI) with an OR = 1,8 (CI95% [1,55-2,62]); and finally age with an OR = 1,2 (CI95% [1,06-2,27]).

The relationship between HT, diabetes and obesity is well elucidated in the literature (Garcia et al. 2008; Gus et al. 2004; Chuang et al. 2006). It is known that obese subjects have higher tension values, associated with a cardiac output and a blood volume so high than the subjects with normal weight (Garcia et al. 2008). In the same context, the pancreas of the obese person secretes more insulin compared to a non obese subject to confront the insulin resistance and counteract high rate of the glycemia (Garcia et al. 2008; Lean et al. 1990; Haslam and James 2005).

This hyperinsulinemia generates many physiological modifications (Schinner et al. 2005), such the increase of the rate of adrenaline and consequently the cardiac output (Lean et al. 1990; Haslam and James 2005), and the narrowing of blood vessels which results in an increase in stiffness and an elevation of blood pressure. On the renal level, hyperinsulinemia promotes the reabsorption of Na + and water, which leads subsequently to an increase of blood volume and the blood pressure (Jansen et al. 2010). The positive effect of the age on HT is known, especially when it is associated with other risk factors such as diabetes and obesity (Renzaho et al. 2014).

## Conclusions

This study has achieved major objectives traced for this work, we have estimated the frequency of HT in the east of Morocco (31.7%), and we have also proved and calculated for the first time the different relationship between HT, type 2 diabetes and obesity in this region. The high frequency of HT and also diabetes and obesity in the east of Morocco require the adoption of a global, urgent and effective strategy by the ministry of health, which must involve all the organizations and interested people of the civil society.
